# SMYD1, an SRF-Interacting Partner, Is Involved in Angiogenesis

**DOI:** 10.1371/journal.pone.0146468

**Published:** 2016-01-22

**Authors:** Xiangli Ye, Yu Qian, Qian Wang, Wuzhou Yuan, Xiaoyang Mo, Yongqing Li, Zhigang Jiang, Wei Xu, Yun Deng, Yongqi Wan, Xiongwei Fan, Xiushan Wu, Yuequn Wang

**Affiliations:** 1 The Center for Heart Development, Key Lab of MOE for Development Biology and Protein Chemistry, College of Life Sciences, Hunan Normal University, Changsha, 410081, Hunan, China; 2 Shanghai Key Laboratory of Regulatory Biology, Institute of Biomedical Sciences and School of Life Sciences, East China Normal University, Shanghai, 200241, China; 3 College of Medicine, Hunan Normal University, Changsha, Hunan, 410013, China; Louisiana State University, UNITED STATES

## Abstract

Previous studies have demonstrated that Smyd1 plays a critical role in cardiomyocyte differentiation, cardiac morphogenesis and myofibril organization. In this study, we uncovered a novel function of Smyd1 in the regulation of endothelial cells (ECs). Our data showed that Smyd1 is expressed in vascular endothelial cells, and knockdown of SMYD1 in endothelial cells impairs EC migration and tube formation. Furthermore, Co-IP and GST pull-down assays demonstrated that SMYD1 is associated with the Serum Response Factor (SRF). EMSA assays further showed that SMYD1 forms a complex with SRF and enhances SRF DNA binding activity. Our studies indicate that SMYD1 serves as an SRF-interacting protein, enhances SRF DNA binding activity, and is required for EC migration and tube formation to regulate angiogenesis.

## Introduction

Angiogenesis in vertebrates is a fundamental and dynamic process for the extension of the vascular network and is associated with tight coordination of endothelial cell (EC) proliferation, sprouting, and migration, and the recruitment of mural cells [1, 2]. Various signaling molecules, such as VEGF, FGF and TGF-β [3–10], play a crucial role in vascular tree development. These signaling pathways exhaust their function in ECs by eliciting an array of biological effects and intracellular signaling events.

SMYD1, also termed Bop1, is highly expressed in skeletal muscle and heart tissues [11]. The SMYD1 protein harbors a MYND domain that functions as a protein-protein interaction domain, and SET domains that commonly act as a methyltransferase for histones or other proteins [12–16]. As a transcriptional co-factor, SMYD1 plays a critical role in cardiac morphogenesis and myofibril organization [17–20]. In mice, deletion of Smyd1 leads to death at embryonic day 10 due to a loss of function of the right ventricle. In zebrafish, blocking Smyd1 protein expression results in the accumulation of blood cells in the yolk during the embryonic stages [21], suggesting novel roles for Smyd1 in the maintenance of vascular integrity. However, the function of Smyd1 in endothelial cells has not been reported in prior studies.

Our previous studies identified SMYD1 as a downstream target of serum response factor (SRF) that plays vital roles in myogenic differentiation. Recently, the function of SRF in angiogenesis has received much attention. Previous studies demonstrate that SRF regulates EC migration, actin polymerization and tip cell morphology during sprouting angiogenesis, mediating VEGF and FGF signaling. During angiogenesis SRF targets several cytoskeletal proteins that are related to cell motility, ECs junction assembly and vascular integrity [22–28]. Therefore, we hypothesized that SMYD1 might play a role in angiogenesis through regulating SRF signaling.

Here, we uncovered a novel function of Smyd1 in angiogenesis. Our data demonstrates that Smyd1 is expressed in vascular endothelial cells, and knockdown of SMYD1 in ECs impairs the migration of ECs and tube formation. Co-IP and GST pull-down assays demonstrate that SMYD1 associates with SRF. EMSA assays suggest that SMYD1 forms a complex with SRF and enhances SRF DNA binding activity. All these data imply that SMYD1 regulates the migration of ECs and tube formation, possibly through interacting with SRF and enhancing SRF DNA binding activity. Together, our findings suggest that SMYD1 serves as an SRF-interacting protein, enhances SRF DNA binding activity, and is required for EC migration and tube formation.

## Results

### SMYD1 is expressed in vascular endothelial cells

**Fig 1:**
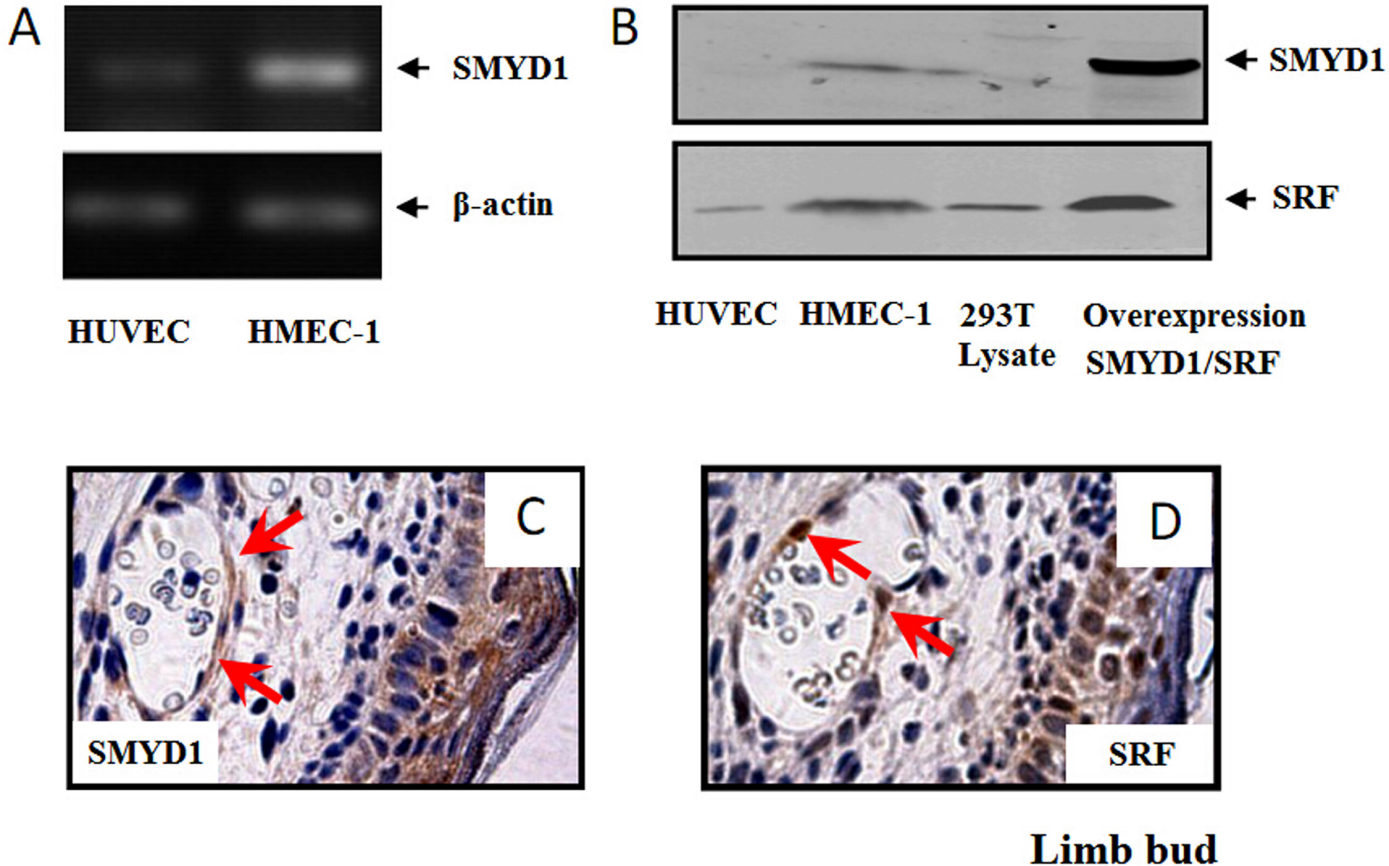
SMYD1 expressed in vascular endothelial cells. (A) RT-PCR analysis of SMYD1 gene expression in HUVEC and HMEC-1 cells. (B) Western blot analysis of endogenous SMYD1 and SRF expression in HUVEC and HMEC-1 cells. Over-expressed Flag-SMYD1 and HA-SRF were used as a positive control to indicate the sizes of SMYD1 or SRF. (C) and (D) Immunohistochemical expression of SRF and SMYD1 in mouse limb buds at E12.5 was shown. Both SMYD1 and SRF were expressed by vascular endothelial cells. Sections were slightly counterstained with hematoxylin.

We first examined SMYD1 expression in vascular endothelial cells. With real-time PCR (RT-PCR) and western blotting assays, we found that SMYD1 is mainly expressed in human dermal microvascular endothelial cells (HMEC-1) (Fig 1A and 1B). Immunohistochemical staining was performed using a special antibody against SMYD1 [11]. SMYD1 positive cells were specifically detected in the ECs of vessels of embryonic limb buds at E12.5 (Fig 1C and 1D).

### Knockdown of SMYD1 impairs the migration of ECs and tube formation

**Fig 2:**
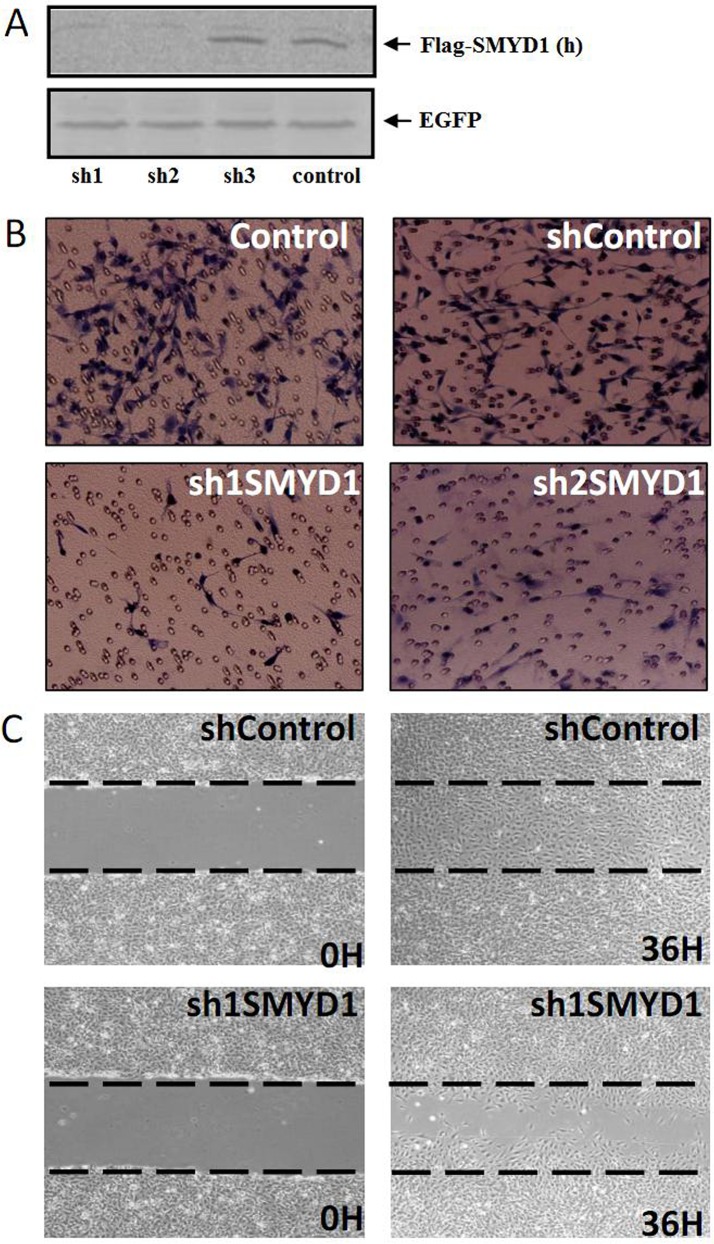
Knockdown of SMYD1 inhibited HMEC-1 cell migration. (A) The efficiency of lentiviral-based shRNA-mediated protein interference. 293T cells were co-transfected with human or mouse Flag-SMYD1 and SMYD1 shRNA (control, sh1, sh2 or sh3) lentivirus vector as indicated. 48 h later, proteins that extracted from these cells were subjected to western blotting. (B) Boyden chamber cell migration assays were performed as described in materials and methods. sh control, vector control, empty vector transfected HMEC-1 cells; sh1SMYD1, sh2SMYD1, sh3SMYD1, and SMYD1 shRNA transfected cells. (C) Monolayer scratch wound healing assay in HMEC-1 cells. Confluent monolayer cells were serum-starved overnight (16 h), and subjected to scratch wounding as described in materials and methods. After 36 h migration, wound closure was photographed under 200× magnification. Dashed lines indicated the edge of the “wound” right after the scratch.

**Fig 3:**
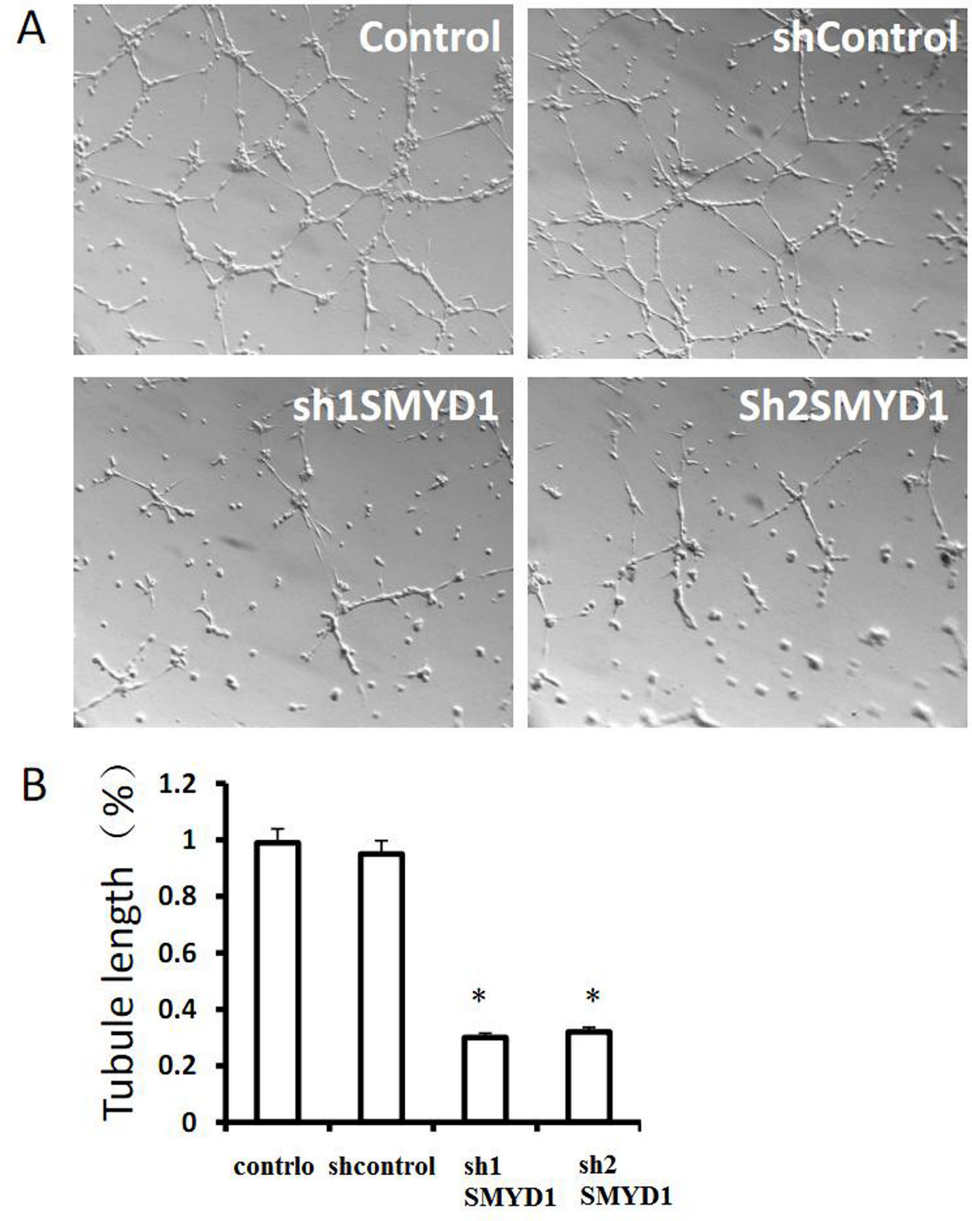
SMYD1 knockdown impairs angiogenesis *in vitro*. (A) and (B) Capillary tube formation of HMEC-1 cells infected with sh control or SMYD1 shRNAS (sh1SMYD1 or sh2SMYD1) seeded onto Matrigel. After 4 to 6 h, HMEC-1 cells were fixed and tubular structure was quantified by calculating the tube length of high-power fields (200×). All error bars represent SD. A value of *p<0.05 was compared with the control.

The expression of Smyd1 in the HMEC-1 cell line and in vessel ECs of embryonic limb buds implies a role for Smyd1 in endothelial cell biology, such as migration and tube formation. To examine the roles of SMYD1 in these processes, we used lentiviral-mediated RNA interference (RNAi) to generate SMYD1 deficient HMEC-1 cells by targeting two independent sequences of SMYD1 mRNA. The efficiency of lentiviral-based shRNA-mediated RNAi was detected by western blotting (Fig 2A). Next, we tested the role of Smyd1 in EC migration using wound healing and Boyden chamber assays. The number of migrated cells in SMYD1 deficient cells decreased significantly compared to the control cells in both of these two migration assays (Fig 2B and 2C, S1 Fig). Tube formation is an important parameter of EC function in angiogenesis. Knockdown of SMYD1 inhibited the tube formation of ECs with decreases in tubule length and in the overall complexity of the network (Fig 3A and 3B). Taken together, these results demonstrated that SMYD1 is a critical regulator that modulates the angiogenic behavior of ECs *in vitro*.

### SMYD1 interacts with SRF

**Fig 4:**
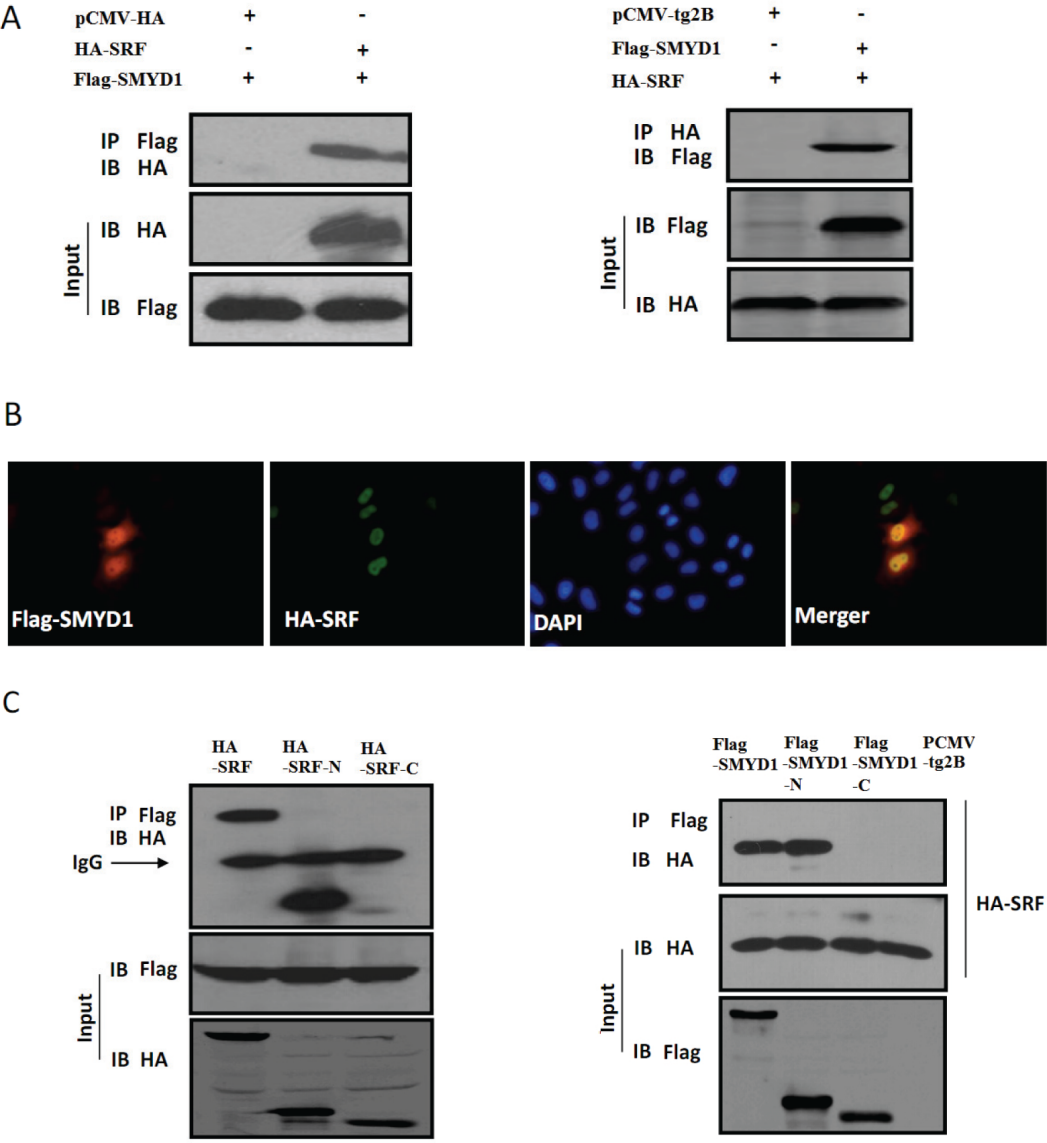
SMYD1 interacts with SRF in cells. (A) Co-IP experiment in 293T cells. Flag-SMYD1, HA-SRF or null vector (pCMV-HA or pCMV-tg2B) were transfected into 293T cells as indicated and cell lysates were then immunoprecipitated using anti-Flag or anti-HA antibody. The immunoprecipitates were examined by western blotting using anti-HA or anti-Flag antibodies. Input represented 10% of cell lysates used in the Co-IP experiment. (B) SMYD1 co-localization with SRF. Hela cells were transiently transfected with Flag-SMYD1 and HA-SRF. Then, cells were fixed and stained for anti-Flag and anti-HA antibodies. (C) and (D) Mapping of SMYD1 and SRF to identify the SRF or SMYD1-binding region. A total of 293T cells were transfected with Flag-SMYD1 in addition to different HA-SRF deletion mutants as indicated, or cells were transfected with HA-SRF and different Flag-SMYD1 deletion mutants as indicated. Cell lysates were immunoprecipitated with anti-Flag antibody. The immunoprecipitates and cell lysates were then analyzed by western blotting separately using anti-HA antibody, anti-flag antibody for Flag-SMYD1 and its deletion mutants as indicated, and anti-HA antibody for HA-SRF and its deletion mutants as indicated.

Previous studies have demonstrated that SRF plays an important role in sprouting angiogenesis and small vessel integrity in mouse embryos [16, 17]. As shown in Fig 1B and 1D, SRF is expressed in cell lines of ECs (HMEC-1 and HUVEC) and in the vessel ECs of the embryonic limb buds at E12.5. In view of the parallel protein expression between SMYD1 and SRF, we speculated that SMYD1 might serve as a SRF partner to regulate EC migration and tube formation. To test this possibility, 293T cells were transfected with SMYD1 and SRF, and then subjected to a co-immunoprecipitation (Co-IP) experiment. We found that SMYD1 physically interacts with SRF (Fig 4A). The nuclear localization of SMYD1 and SRF was detected by immunostaining, which further indicated that SMYD1 interacts with SRF (Fig 4B). To identify the SRF-association domain of SMYD1 or the SMYD1-association domain of SRF, Co-IP experiments were performed using 293T cells transiently transfected with SMYD1 and SRF-N or the SRF-C terminus domain (Fig 4C) or transfected with SRF and different SMYD1 deletion mutants (Fig 4D). The results demonstrated that the SRF N-terminus domain and the SMYD1 N-terminus domain were important for protein interaction. Together, these results suggest that SMYD1 associates with SRF.

**Fig 5:**
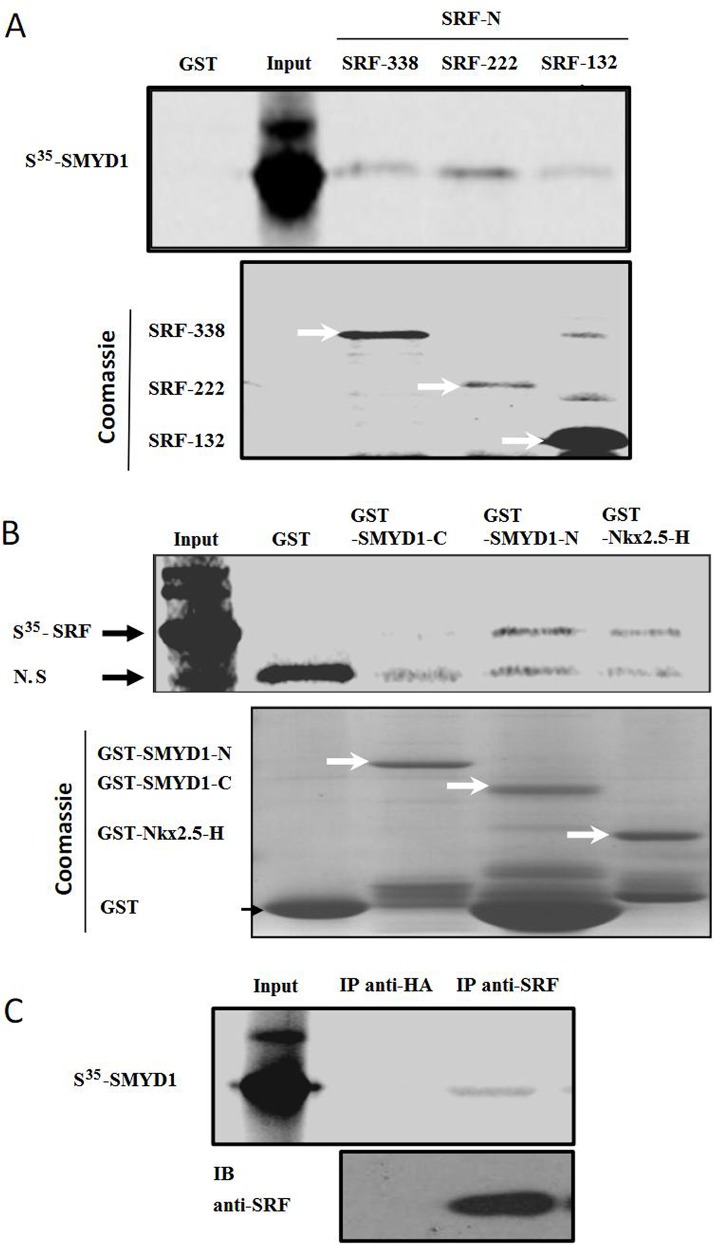
SMYD1 interacts with SRF *in vitro*. GST pull-down assay for determination of SMYD1-SRF interaction. (A) The GST control protein, GST-SRF and its deletion mutants were incubated with SMYD1 protein that was produced by *in vitro* translation in the presence of [^35^S] methionine. Bound proteins were analyzed by autoradiography. (B) GST control protein, GST-SMYD1-C, GST-SMYD1-N and GST-Nkx2.5-H (positive control) were incubated with SRF protein, produced by *in vitro* translation in the presence of [^35^S] methionine. Bound proteins were analyzed by autoradiography. (C) SMYD1 protein that was produced by *in vitro* translation in the presence of [^35^S] methionine. SRF protein, which was produced by *in vitro* translation in the absence of [^35^S] methionine. The Co-IP experiment was carried out as described in the Materials and Methods section. Anti-HA antibody (negative control) and anti-SRF antibody were used for IP, and the immunoprecipitates were examined by autoradiography. The results represent the averages ± mean from three independent experiments.

To further demonstrate that SMYD1 interacts with SRF *in vitro*, GST-SRF and its deletion mutants were incubated with SMYD1 protein that was produced by *in vitro* translation in the presence of [^35^S] methionine, in which bound proteins were analyzed by autoradiography. As shown in Fig 5A, [^35^S] methionine-labeled SMYD1 bound the bacterially expressed GST-SRF-N (SRF-338, SRF-222 and SRF-132) fusion protein, but not to GST alone. [^35^S] methionine labeled SRF bound to the bacterially expressed GST-SMYD1-N fusion protein and to GST-Nkx2.5-H (positive control), but not to GST alone or to the GST-SMYD1-C fusion protein, as shown in Fig 5B. The SMYD1-SRF interaction was further investigated by a Co-IP experiment *in vitro*. As shown in Fig 5C, [^35^S] methionine labeled SMYD1 was also found to interact with no-[^35^S] methionine labeled SRF.

In summary, all of these findings strongly suggest that SMYD1 interacts with SRF.

### SMYD1 forms a complex with SRF and enhances SRF DNA binding activity

**Fig 6:**
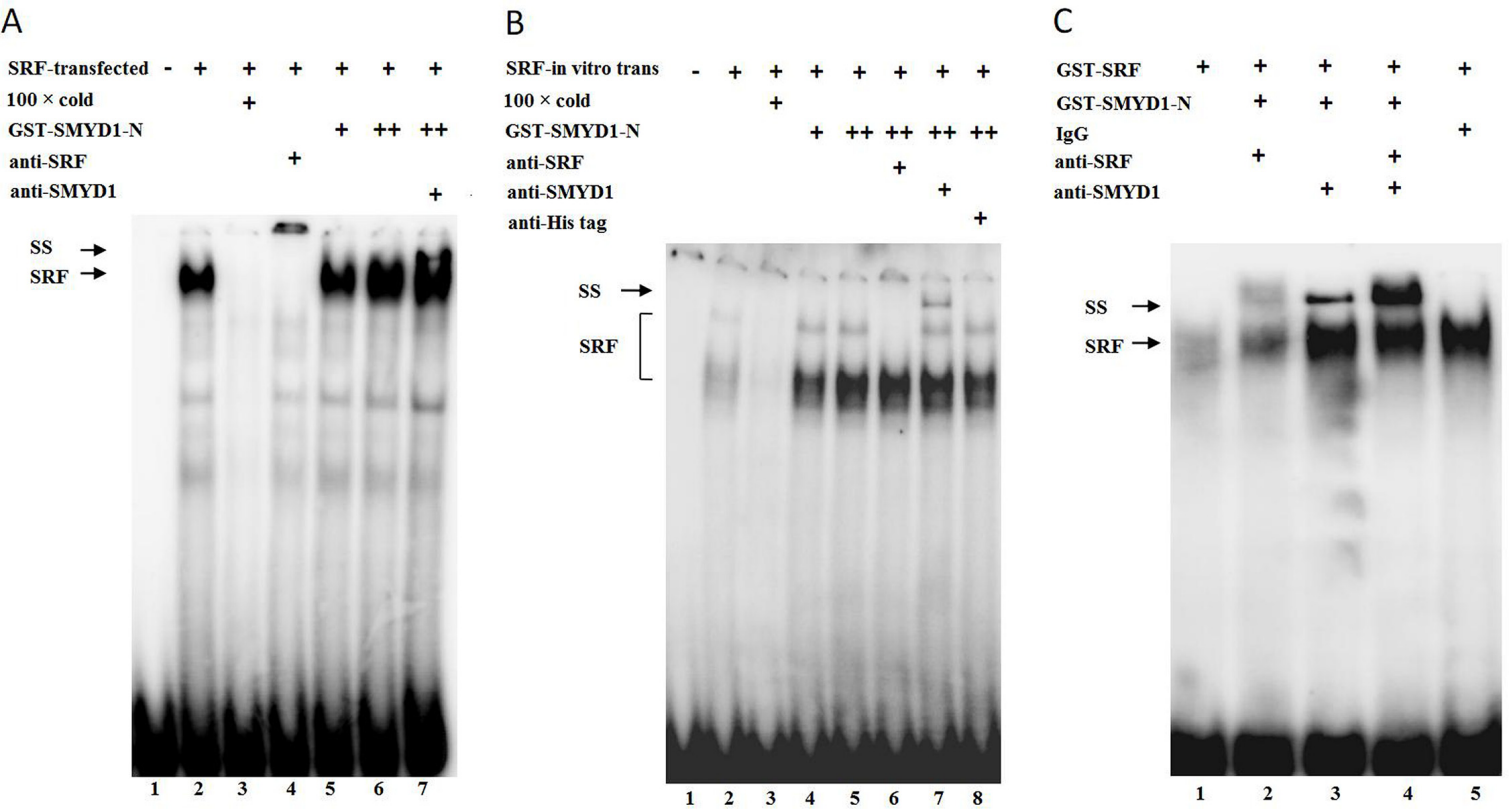
SMYD1 forms complex with SRF and enhances SRF DNA binding activity. Nuclear protein was isolated from 293T cells. (A) EMSA was performed using purified GST-SMYD1-N fusion protein, SRF- *in vitro* trans-protein and ^32^P-labeled oligonucleotide probes containing a consensus binding motif for SRF. Lane 1 was vector control, lane 3 contained 100× cold as a competitor. SMYD1 dose-dependently enhances SRF DNA binding activity (lanes 2, 5, 6). The anti-SRF antibody (lane 4) and anti-SMYD1 antibody (lane 7) were used for the supershift assay. (B) EMSA was performed using purified GST-SMYD1-N fusion protein, SRF-transfected-protein and ^32^P-labeled oligonucleotide probes containing a consensus binding motif for SRF. Lane 1 was vector control, lane 3 contained 100× cold as a competitor. SMYD1 dose-dependently enhances SRF DNA binding activity (lanes 4, 5). The anti-SRF antibody (lane 6) and anti-SMYD1 antibody (lane 7) were used for the supershift assay. The anti-His antibody was used as a control (lane 8). (C) EMSA was performed using purified GST-SMYD1-N fusion protein, SRF fusion protein and ^32^P-labeled oligonucleotide probes containing a consensus binding motif for SRF. The anti-SRF antibody (lane 2, 4) and anti-SMYD1 antibody (lane 3) were used for the supershift assay. The IgG was used as a control (lane 5).

Smyd1 is recruited as a transcriptional co-activator [20] and is not able to bind DNA directly. It is likely recruited to tissue-specific targets through physical interactions with other DNA binding partners. SRF often forms regulatory complexes with other transcriptional regulators through its DNA binding domain. However, whether SMYD1 is recruited to form a complex with SRF to activate SRF binding protein remains unclear. To investigate this issue, electrophoretic mobility shift assays (EMSA) were performed using synthetic probes identical to the CArG site found in the SRF promoter. Full-length SRF constructs were transfected into 293T cells. Nuclear extracts from the transfected cells were harvested, and EMSAs were performed. As shown in Fig 6A, the intensity of the binding complex band was increased with increased amounts of purified GST-SMYD1-N protein (Fig 6A, lane 5 and lane 6). Moreover, the complex was shifted with the addition of the special SMYD1 antibody (Fig 6A, lane 7), indicating that SMYD1 forms a complex with SRF and increases SRF DNA binding activity. Similar EMSA assays were performed using *in vitro* translated SRF protein or purified GST-SRF protein. As shown in Fig 6B, SRF DNA binding activity was enhanced with the increased amount of purified GST-SMYD1-N protein (Fig 6B, lane 4 and lane 5) and supershifts were also observed (Fig 6B, lane 7; Fig 6C, lane 3 and lane 4). Taken together, these results strongly suggest that SMYD1 forms a complex with SRF and enhances SRF DNA binding activity.

## Discussion

In this study, our results reveal a novel role for SMYD1 in modulating EC migration and tube formation *in vitro*. We further demonstrate that SMYD1 forms a complex with SRF and enhances SRF DNA binding activity. Thus, our data indicate that SMYD1 might regulate EC migration and tube formation through interacting with SRF to regulate SRF DNA binding activity during angiogenesis.

### SMYD1 is required for EC migration and tube formation

Angiogenesis depends on the polarization, motility and migration of ECs, which requires cell cytoskeleton proteins [1, 2]. SMYD1 regulates myocardial cell differentiation as a downstream effect of MEF2C signaling [18]. Our previous study also demonstrated that SMYD1 mediated the regulation of major muscle regulatory factors, SRF and myogenin regulated cytoskeleton proteins, which are involved in myogenic differentiation [11]. As a transcript co-factor in heart tissues and skeletal muscle [17–20], the functions of SMYD1 in other tissues were rarely studied. In zebrafish, inactivation of Smyd1 induced accumulation of hemocyte in the yolk during the embryo stages, which suggests novel roles for Smyd1 in the maintenance of vascular integrity [21]. In addition, the potential function of SMYD1 in cytoskeleton proteins might be involved in cavitation of angiogenesis and pseudopodia stretching of ECs. As expected, the expression of SMYD1 was found in ECs of early mouse embryos, and abundant SMYD1 protein was present in the HMEC-1 cell cultured *in vitro*, indicating an autonomous function of Smyd1 on the ECs. However, a previous analysis of expression in all tissue extracts could not accurately reflect the expression of Smyd1 in a single cell type because the vessel distribution was present in both the cardiac and skeletal muscle tissue. Further data show that knockdown of SMYD1 noticeably reduces EC migration and tube formation, indicating that SMYD1 is required for the migration of ECs and tube formation *in vitro*. Therefore, we identified the *Smyd1* gene as a regulation factor for vascular endothelial cell movement that might be involved in angiogenesis.

### SMYD1 serves as an SRF-interacting protein and enhances SRF DNA binding activity

SRF is an evolutionarily conserved transcription factor that regulates cell growth, migration, cytoskeletal organization, cardiomyocyte differentiation and myogenesis. The MADS box of the SRF protein binds to a consensus DNA sequence known as the CArG-box [5'-CC(A/T)6GG-3'] within the promoter of downstream target genes [22–26]. In recent years, research has shown that SRF plays a significant role in each phase of angiogenesis, including EC differentiation into tip cells and stalk cells, tip cell sprouting and integrity remodeling [27, 28]. SRF regulation of cell filopodia can be achieved via the upstream regulation of GTPase-signal-target-cytoskeleton protein and actin, while SRF regulation of vessel integrity is achieved through its downstream regulation of cell-cell adhesion molecules (VE-Cad, ZO-1) of the blood vessel endothelium [28]. Cell adhesion and cell motility seem to be paradoxical functions for the same protein. A reasonable explanation for this activity might be that the different effects of SRF are dependent on its interactions with different transcriptional co-activators that are expressed simultaneously [29]. Because different protein-protein interactions in the nucleus can adjust the appetency of SRF toward its target gene and activate or inhibit the transcriptional activity of SRF as well, unveiling the specific roles of SRF at different stages of angiogenesis might lead to the discovery of a novel interacting protein. Here, we identified a new molecule that mediates SRF regulation of angiogenesis. Our Co-IP and GST pull-down assays demonstrated that the N-terminal of SMYD1 interacts with the 133–338 segment of the SRF protein, which contains a MADS box (an area where SRF binds DNA and regulates transcription). Cell fluorescence co-localization shows that both SRF and SMYD1 are co-located in the nucleus, and embryonic immunohistochemistry assays demonstrate that there is a similar distribution of SMYD1 and SRF in the blood vessel endothelium. The results of the EMSA indicate that SMYD1 can form a ternary complex with SRF and its target DNA and that SMYD1 can also enhance the appetency of SRF toward its target DNA. In addition, an *in vitro* cell function analysis demonstrates that both the migration of ECs and the inhibition of vessel generation caused by knockdown of SMYD1 mimic the phenotype induced by SRF knockdown in ECs [27]. Therefore, SMYD1 should be an important co-factor of angiogenesis because it regulates the migration of ECs; the interaction of SMYD1 and SRF can endow SRF with a stronger appetency for binding more target genes or enhance the transcriptional activation of intrinsic target genes.

In conclusion, our results demonstrate that SMYD1 is an SRF-interacting protein that plays a crucial role in angiogenesis through forming a complex with SRF and regulating SRF DNA binding activity. The present study uncovers a new function of SMYD1 in ECs, which sheds new light on biological significance of SMYD1.

## Materials and Methods

### Mice

This study received approval from the Animal Ethics Committee and the Medical Ethics Committee of Hunan Normal University and East China Normal University. The mouse experiments were performed in accordance with the Institutional Guidelines for the Care and Use of Laboratory Animals (National Research Council, 1996) and Animals Act (China, 2006).

### Cell culture and transfection

293T cells were grown in DMEM as described previously [11]. Cells were transfected with Lipofectamine 2000 (Invitrogen) according to the manufacturer's suggestions. Human dermal microvascular endothelial cells (HMEC-1) and Human umbilical vein endothelial cells (HUVEC) [30, 31], which have endothelial characteristics of cell migration and tubular structure formation, were purchased from Science Research Laboratories (ScienCell Research Laboratories, San Diego, CA, USA) and stored by the lab of Institute of Biomedical Sciences and School of Life Sciences, East China Normal University. HMEC-1 cells were maintained in MCDB-131 medium (Invitrogen, Life Technologies) supplemented with 10% fetal bovine serum (FBS), 10 ng/mL of human epidermal growth factor (EGF) (Sigma, St. Louis, MO), 1 μg/mL of hydrocortisone, 5 mg/mL of L-glutamine, and antibiotics (penicillin-streptomycin) at 37°C with 5% CO_2_.

### Immunohistochemistry

Tissues were obtained, fixed and embedded with paraffin according to standard procedures. Anti-SMYD1 (1:200) and anti-SRF (1:500) were used to stain limb bud vessels in 5-μm sections. After staining with DAB (brown precipitate). Slides were counterstained with hematoxylin. A Leica DM 4000B photomicroscope was used to take the images.

### RT-PCR analysis

Total RNA was extracted from cells by using TRIzol reagent (Invitrogen) and 1 μg of total RNA was reverse-transcribed in a 20 μL reaction solution according to the manufacturer's protocol using the PrimeScriptTM RT Reagent Kit (TakaRa). PCR products were resolved in a 1.2% agarose ethidium bromide gel and visualized with ultraviolet light.

### Lentivirus-based knockdown

The virus-based knockdown was conducted using the pll3.7 vector. The shRNA oligos targeting human SMYD1, GTTGATGGCTACATGAAA (Sh1) and GTGAAGGAGATGATACAA (Sh2), were subcloned into modified pll3.7 in which the GFP was substituted with DsRed using the Nhe I and EcoR I sites. Viral production involved transfecting 293T cells with overexpression, knockdown, or control construct together with viral packaging vectors (psPAX2, pMD2G) using calcium phosphate transfection. The virus was harvested from the supernatant 48 h post-transfection before being used to infect target cells (2 × 10^5^) with 5 × 10^6^ TCID50. High titer virus was prepared by ultracentrifugation at 1.5 × 10^5^ g at 4°C before being resolved in PBS.

### Monolayer wound-healing experiments

HMEC-1 cells were seeded in 6-well plates that were precoated with 0.1% gelatin (Sigma) in MCDB131 medium containing 10% FBS. After growing to full confluence, HMEC-1 cells were incubated with 10 μg/mL of mitomycin C and inactive cells at 37°C, 5% CO_2_ for 2.5 h. The cells were scraped with a cross in the middle of the well using a 200 μL pipette tip. After washing with PBS, MCDB131 medium supplemented with 0.5% FBS was added into the wells. Images were taken after 36 h of incubation at 37°C and 5% CO_2_. The experiments were performed in triplicate and repeated at least 3 times.

### Boyden chamber cell migration assay

The Transwell (Millipore, Inc.) was coated with 0.1% gelatin for 30 min in a cell incubator. HMEC-1 cells (100 μL, 40,000 cells) were added into transwell filter chambers (the inserts) of the Boyden chamber system. The bottom chambers were filled with MCDB-131 media with 0.2% FBS. After 24 h of migration, the cells on the top surface of the membrane (non-migrated cells) were scraped with a cotton swab to the bottom sides. The cells on the bottom sides of the membrane (invasive cells) were incubated with cold 4% paraformaldehyde for 30 min, then migrated cells were stained with crystal violet. Images were taken with an Olympus inverted microscope and invasive cells were quantified by manual counting.

### Tube formation assays

Matrigel was thawed at 4°C, and each well of the pre-chilled 24-well plates was coated with 200 μL of Matrigel and incubated at 37°C for 45 min. Then, HMECs were added in 500 μL of culture medium (50,000 cells). After 4 to 6 h of incubation at 37°C and 5% CO_2_, endothelial cell tubular structure formation was quantified by calculating the tube length of high-power fields (HPFs; 200×) with an Olympus inverted microscope.

### Western blot analysis

Cells were lysed and the resulting supernatant was loaded in a sodium dodecyl sulfate polyacrylamide gel electrophoresis (SDS-PAGE) gel as described previously [11]. The antibodies used were anti-SMYD1 (Abcam, 1:1,000), anti-SRF (Santa Cruz, 1:2,000), and anti-GFP (Abcam, 1:1,000). Specific IRDye® 800CW conjugated goat (polyclonal) anti-rabbit IgG was used to detect protein expression with an Odyssey infrared imaging system (LI-COR Bioscience, U.S.A.).

### Electrophoretic mobility shift assays

Cells were cultured in six-well plates in DMEM containing 10% FBS and then harvested by manual scraping as described previously [11]. Human promoter-derived SRF binding site oligonucleotides were synthesized, annealed and labeled with γ^32^-P as described previously [11]. SRF- and SMYD1-specific antibodies were then incubated in appropriate reactions for 20 min on ice. DNA-protein complexes were then resolved on 5% PAGE gels and protein DNA complexes were visualized as described previously [11].

## Supporting Information

S1 Fig Knockdown of SMYD1 inhibited HMEC-1 cell migration. (A) Quantification analysis of the relative cells number in Boyden chamber cell migration assay. *p< 0.05; n = 3. (B) The migrated distance of the wound edge in HMEC-1 cells was quantified. *p< 0.05; n = 7
(TIF)
