# Supplementary figures and images for: SMYD1, an SRF-Interacting Partner, Is Involved in Angiogenesis
Source: PLoS One. 2016 Jan 22;11(1):e0146468. doi: 10.1371/journal.pone.0146468 (PMC4723226; doi:10.1371/journal.pone.0146468)

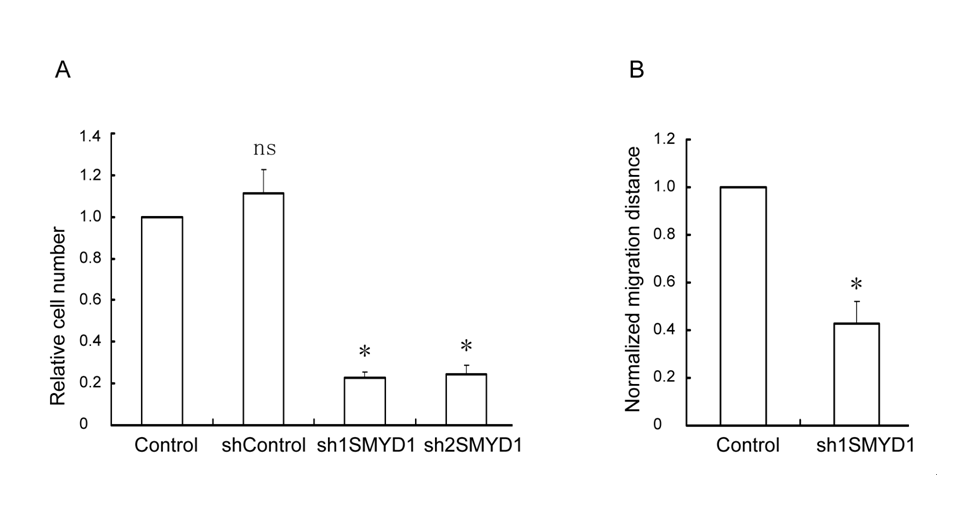

Supplement: S1 Fig — (A) Quantification analysis of the relative cells number in Boyden chamber cell migration assay. *p< 0.05; n = 3. (B) The migrated distance of the wound edge in HMEC-1 cells was quantified. *p< 0.05; n = 7 (TIF) [file pone.0146468.s001.tif]
